# Beta-Secretase 1 Recruits Amyloid-Beta Precursor Protein to ROCK2 Kinase, Resulting in Erroneous Phosphorylation and Beta-Amyloid Plaque Formation

**DOI:** 10.3390/ijms241310416

**Published:** 2023-06-21

**Authors:** István Hajdú, Barbara M. Végh, András Szilágyi, Péter Závodszky

**Affiliations:** 1 Institute of Enzymology, Research Centre for Natural Sciences, 1117 Budapest, Hungary; vegh.barbara@ttk.hu (B.M.V.); szilagyi.andras@ttk.hu (A.S.); 2 Faculty of Information Technology and Bionics, Pázmány Péter Catholic University, 1083 Budapest, Hungary

**Keywords:** rho-kinase (ROCK), phosphorylation, amyloid precursor protein (APP), beta-secretase (BACE1), allosteric binding site

## Abstract

The amyloidogenic processing of APP depends on two events: its phosphorylation by ROCK2 (at Thr654) and the phosphorylation of the APP-cleaving enzyme BACE1 (at Ser498). However, the mechanisms and structural details of APP-ROCK2 and BACE1-ROCK2 binding are unknown. Using direct physical methods in combination with an in silico approach, we found that BACE1 binds into the substrate-binding groove of ROCK2 with a low affinity (K_d_ = 18 µM), while no binding of APP to ROCK2 alone could be detected. On the other hand, a strong association (K_d_ = 3.5 nM) of APP to the weak ROCK2-BACE1 complex was observed, although no stable ternary complex was detected, i.e., BACE1 was displaced by APP. We constructed a sequential functional model: (1) BACE1 weakly binds to ROCK2 and induces an allosteric conformational change in ROCK2; (2) APP strongly binds to the ROCK2-BACE1 complex, and BACE1 is released; and (3) ROCK2 phosphorylates APP at Thr654 (leading to a longer stay in the early endosome during APP processing). Direct fluorescence titration experiments showed that the APP_646–664_ or APP_665–695_ fragments did not bind separately to the ROCK2-BACE1 complex. Based on these observations, we conclude that two binding sites are involved in the ROCK2-APP interaction: (1) the substrate-binding groove, where the APP_646–664_ sequence containing Thr654 sits and (2) the allosteric binding site, where the APP_665–695_ sequence binds. These results open the way to attack the allosteric site to prevent APP phosphorylation at Thr654 by ROCK2 without inhibiting the activity of ROCK2 towards its other substrates.

## 1. Introduction

Alzheimer’s disease is currently the leading cause of dementia, with no effective treatment. Evidence indicates that the amyloid precursor protein (APP) and its derivative, amyloid-β (Aβ) peptide, play an important role in its development [1]. Although Aβ cannot account for all features of Alzheimer’s disease, reducing Aβ production or accumulation is a major therapeutic strategy to confront the disease [2]. The accumulation of Aβ peptides in senile plaques is the consequence of pathological processing of APP. There are two major pathways of APP processing: a non-amyloidogenic pathway involving α-secretase [3], and an amyloidogenic pathway involving the primary cleavage of APP by β-secretase [4]. Both pathways include the subsequent action of γ-secretases [5]. The relative contribution of each pathway to the whole processing mainly depends on the subcellular localization of the process: the amyloidogenic pathway typically occurs in the early endosome [6], where the acidic environment favors the proteolytic activity of β-secretase, also known as β-site APP-cleaving enzyme 1 (BACE1), accounting for the β-cleavage. The APP protein (like other proteins) undergoes trafficking between the different subcellular components. It was shown that phosphorylation of the intracellular domain of the APP modifies its trafficking behavior [7]. The phosphorylation of Thr654 by ROCK2 extends the residence time of APP in the early endosome, which leads to a higher probability of Aβ formation. Interestingly, it was also demonstrated that the phosphorylation of BACE1 at Ser498 by ROCK2 also prolongs its residence time in the early endosome. Based on these results, it is assumed that pharmacological inhibition of ROCK2 could suppress Aβ production by interfering with two different pathways in parallel. It was demonstrated that specific inhibition of ROCK2 using an isoform-selective small molecule (SR3677) diminished the production of Aβ in an AD mouse brain via the two mechanisms [8]. Although this molecule leaves ROCK1 activity unmodified [9], the vast number of ROCK2 functions make this kind of specific but activity-targeted inhibition prone to side effects [10].

**Figure 1:**
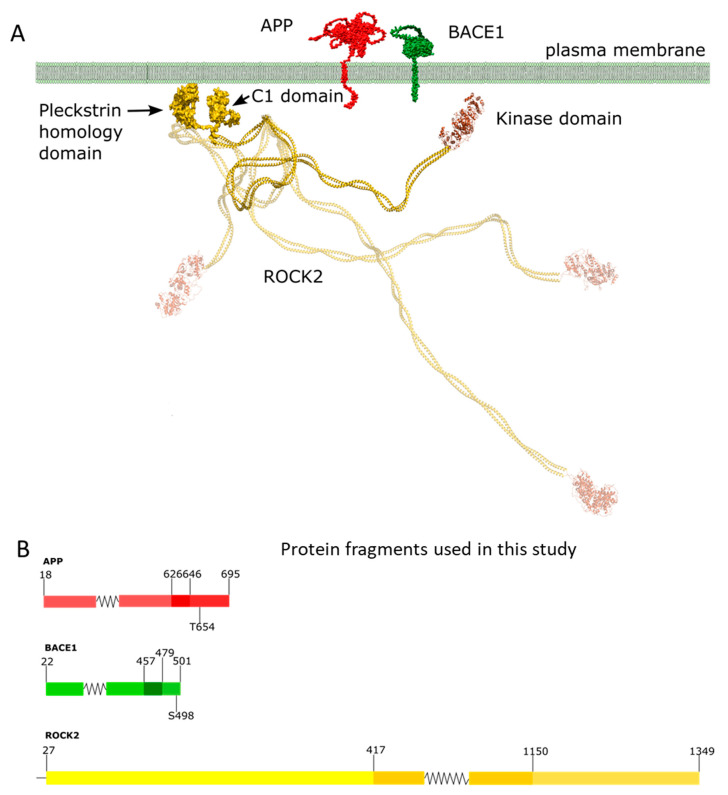
Domain structure, membrane topology and cellular localization of ROCK2, BACE1 and APP. ROCK2 is anchored to the plasma membrane by its C-terminal region, through its split pleckstrin domain bisected by a C1 domain. Both BACE1 and APP are transmembrane proteins with short (23 and 50 residues, respectively) intracellular C-terminal domains (**panel A**). In (**panel B**), we show the expressed protein fragments; in the case of APP, the C-terminal intracellular domain from 646 to 695 was the largest fragment, which comprises the phosphorylatable Thr654. The peptide representing the BACE1 C-terminal intracellular domain (residues 479–501) was used. In the case of ROCK2, the full-length version and the kinase domain (residues 27–417) was used. Schemes follow the same formatting. (**Panel A**) is a modification of our previous image [13], while panel B was edited using Inkscape 1.2 (Inkscape Project. (2020). Inkscape. Retrieved from https://inkscape.org, accessed on 13 May 2023).

ROCK2 is a large, 1388-residue-long multidomain dimeric serine/threonine kinase localized in the cytoplasm of cells anchored to the plasma membrane by their C-terminal regions [11] (Figure 1). The C-terminal region contains a lipid-binding split pleckstrin homology domain which is bisected by an internal cysteine-rich zinc-finger domain. The center of the molecule forms a long coiled-coil domain containing the Rho-binding domain. The catalytic (kinase) domain is located at its N-terminus. Although the structure of the whole molecule is only known at low resolutions [12], it is evident that for enzymatic activity, only the kinase domains are required. Recently, we constructed a functional model to explain the membrane-proximal and membrane-distal activities of ROCK2, where the weak complex between its terminal domains can be dissociated by natural substrates, allowing the kinase domain to access the substrate [13]. The concept of the present study is based on this model.

APP is a type I integral membrane glycoprotein widely expressed both in neuronal and non-neuronal cells having at least three isoforms with different lengths [14]. In the brain, the amyloidogenic 695-residue-long APP_695_ is the most prominent isoform [15], which is preferentially processed to generate Aβ. In the amyloidogenic pathway, APP is first cleaved by BACE1, releasing the soluble ectodomain region of APP (sAPPβ) [16]. The remaining 99-residue C-terminal fragment is cleaved at the ε-site by γ-secretase, releasing the 50-residue-long APP intracellular domain [17] and the Aβ fragment; Aβ is then further processed at the ζ- [18] and γ-sites [19] by γ-secretase to yield the Aβ_40_ and Aβ_42_ final products. The intracellular domain of APP contains a number of phosphorylation sites (Tyr653 [20], Thr654 [8], Ser655 [21,22], Thr668 [23,24,25,26,27], Ser675 [28], Tyr682 [29,30], Thr686 [20] and Tyr687 [31]), which are all phosphorylated to various degrees in the brain of patients with Alzheimer’s disease in contrast to healthy brains, suggesting that the phosphorylation events may have a significant impact on the physiological function of APP.

BACE1 is an aspartyl protease of the pepsin family. BACE1 is a type I transmembrane protein, having an extracellular protease domain, a short transmembrane domain and a short 23-residue cytoplasmic C-terminal domain [32]. BACE1 is widely expressed in the brain, particularly in neurons, oligodendrocytes and astrocytes. At the subcellular level, BACE1 localizes on the plasma membrane and in the endosomal compartments, and has been detected in healthy synaptic terminals and dystrophic neurites surrounding amyloid-β plaques [33]. Post-translational modifications of BACE1 are known; its phosphorylation (mainly at Ser498) is important for cellular trafficking [34], although exactly how the phosphorylation of Ser498 modifies the trafficking pathways is controversial [8,35].

In our study, we focus on the mechanism of APP and BACE1 binding by ROCK2, and the aim is the localization and mapping of the protein–protein interaction sites between these proteins to create the basis for the design or selection of allosteric inhibitors targeting Aβ production.

To reveal the structural features of the interaction, we produced the relevant domains of ROCK2, APP and BACE1, either in recombinant or synthetic forms. Using biophysical methods (including surface plasmon resonance and fluorescence resonance) and structural modeling, we identified the protein–protein interactions and constructed a model for the mechanism of cooperation between these three proteins.

The accumulation of Aβ-peptides in senile plaques—observed in Alzheimer’s disease—is the consequence of erroneous processing of APP. The cause of this is the erroneous phosphorylation of APP and BACE1 by ROCK2. Our goal is to reveal the mechanistic and structural features of the phosphorylation process of BACE1 and APP by ROCK2.

## 2. Results

### 2.1. BACE1-ROCK2 Binary Interaction

**Figure 2:**
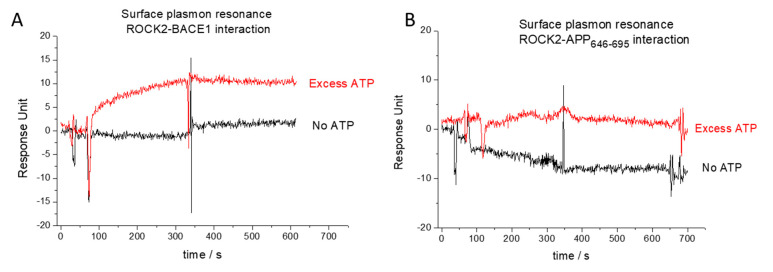
Surface plasmon resonance analysis of binary interaction between ROCK2 and its ligands. Weak interaction was detected between ROCK2 and BACE1 only in the presence of excess ATP (red curve on (**panel A**)), while this interaction was not observed without ATP (black curve on (**panel B**)). No binary interaction was detected between ROCK2 and APP_646–695_, either in the presence (red curve on (**panel B**)) or absence of ATP (black curve on (**panel B**)).

We used surface plasmon resonance to examine the binary binding properties between ROCK2 and its partners. Full-length ROCK2 protein was immobilized on a chip and used as the ligand using a high-density surface of 1700 resonance units. The binding of the BACE1 cytoplasmic domain (BACE1CD) to ROCK2 was only detected at a significant 15-fold molar excess of ATP (Figure 2A). Considering the molecular weights of the ligand (ROCK2) and the analyte, the signal (11 resonance units, RUs) was approximately 50% of the theoretical signal (23 RUs, based on the molecular weight ratio of the analyte and the ligand and the quantity of the immobilized ligand), which implies that half of the bound ROCK2 molecules bind BACE1. As amine coupling is a random binding method, the 50% binding is near the maximum possible signal expected. The exact binding parameters could not be determined, primarily due to the low signal and the high ligand density.

**Figure 3:**
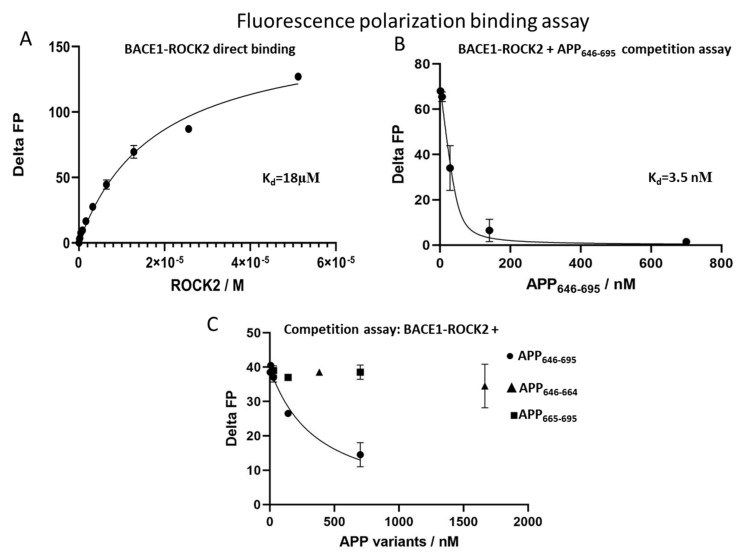
Detection of interactions by fluorescence polarization. Direct titration of fluorescein-labeled BACE1 with ROCK2 showed a binding constant of 18 µM (**panel A**). Competition of the ROCK2-BACE1 interaction with APP_646–695_, showing an interaction constant of 3.3 nM using a fixed BACE1 concentration of 50 nM (**panel B**). The fragments APP_646–664_ (triangles) and APP_665–695_ (squares) do not displace BACE1 from ROCK2, only doing so for APP_646–695_ (circles) (**panel C**).

To quantify the binding affinity between ROCK2 and BACE1, fluorescence polarization measurements were used. The affinity of the binding between either the kinase domain or full-length ROCK2 and fluorescein-labeled C-terminal BACE1 peptide was calculated from a direct measurement using a known concentration (50 nM) of labeled BACE1 and a dilution series for ROCK2. The calculated dissociation constant (K_d_) was 18 µM (Figure 3A) for both the kinase domain and the full-length ROCK2. This indicates that there is a direct interaction between BACE1 and ROCK2, but the binding is weak and is independent of the N-terminal region.

### 2.2. APP-ROCK2 Binary Interaction

Using the same approaches as for ROCK2-BACE1 binding, no binding could be detected between ROCK2 and APP. Surface plasmon resonance experiments did not show binding either with or without ATP added (Figure 2B). In the fluorescence polarization studies, we used C-terminal TAMRA (5-Carboxytetramethylrhodamine)-labeled APP_646–695_ peptides. The use of TAMRA was useful, as its polarizable fluorescence spectrum is readily distinguishable from that of fluorescein. The polarization signal was unchanged, even using the highest available concentration (6 µM) of ROCK2, indicating the lack of direct binary interactions between ROCK2 and APP.

### 2.3. In Search of a ROCK2-BACE1-APP Ternary Interaction

Our finding that the C-terminal domain of APP did not bind to ROCK2 is in contradiction with previous studies [8], indicating that ROCK2 can phosphorylate full-sized APP. To resolve this problem, we assumed that an extra partner is required for the interaction, and the most relevant interaction partner might be BACE1 itself. Since we know that BACE1 and APP are in close proximity in cells [36], we turned to investigating the ternary interactions between ROCK2, BACE1 and APP_646–695_.

As the binding of fluorescein-labeled BACE1 to ROCK2 can easily be monitored in fluorescence polarization assays, we designed a competition fluorescence polarization assay using fixed amounts of ROCK2 and labeled BACE1. When adding APP_646–695_ to the ROCK2-labeled BACE1 mix, a high-affinity interaction with a Kd of 3.5 nM was found (Figure 3B).

We have shown that the 646–695 sequence of APP binds to ROCK2 in the presence of BACE1. It is known that APP_646–695_ is composed of an unstructured stretch (APP_646–664_) and a globular domain (APP_665–695_) [37]. For a more detailed structural mapping of the APP binding site on ROCK2, we studied the binding of both the APP_646–664_ and the APP_665–695_ fragments. The competition assay against ROCK2-BACE1 revealed that neither fragment of APP_646–695_ was capable of displacing BACE1 from ROCK2 (Figure 3C). This result indicates that the full 50-residue segment of APP is required for high-affinity binding to the ROCK2/BACE1.

**Figure 4:**
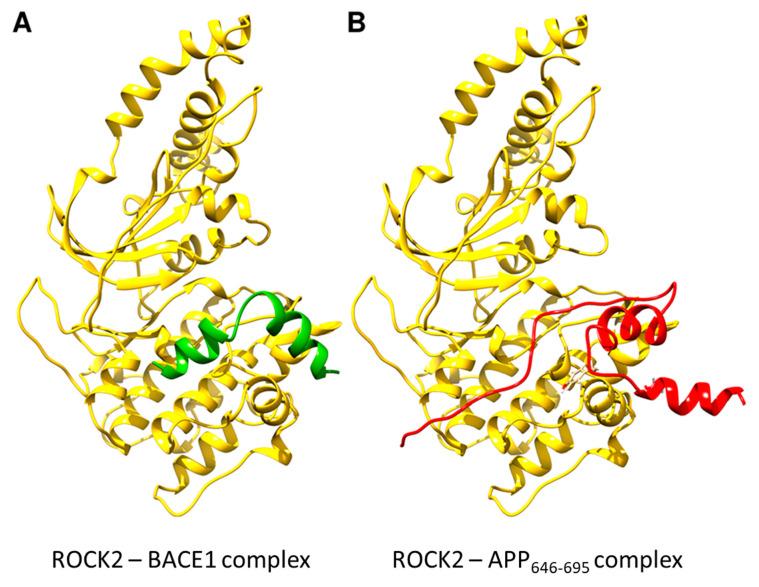
Structures of ROCK2-BACE1 (**A**) and ROCK2-APP_646–695_ (**B**) complexes as predicted using AlphaFold-Multimer. ROCK2 is shown in yellow, BACE1 in green and APP_646–695_ in red. The binding site for both BACE1 and APP is composed of mainly the canonical kinase substrate-binding groove, with the C-terminal tail of APP_646–695_ making contacts with two additional helices on the ROCK2 kinase domain. Structures of ROCK2-BACE1 and ROCK2-APP 646–695 complexes were predicted using AlphaFold-Multimer [39] as implemented in ColabFold [40].

To identify the binding position of ligands on ROCK2, the structures of the binary complexes were modeled with AlphaFold [38]. The binding site for both BACE1 and APP is composed mainly of the canonical kinase substrate-binding groove (Figure 4), with the C-terminal tail of APP_646–695_ making contacts with two additional helices on the ROCK2 kinase domain. Both ligands appear to occupy the binding groove; therefore, a ternary complex between the three proteins is improbable, as was proved experimentally.

## 3. Discussion

Despite decades of intensive research, there is no efficient drug to prevent or cure Alzheimer’s disease. The amyloid hypothesis of the disease suggests that inhibiting Aβ production could reduce symptoms and slow disease progression. During the processing of APP, its cleavage by BACE1 is the rate-limiting step [16]; therefore, the inhibition of BACE1 could be a trivial solution. The development of small molecular inhibitors against BACE1 was set back with a series of unwanted side effects [41]. Previously, it was shown that pharmacological inhibition of ROCK2 activity diminishes sAPPβ production as well as Aβ levels [8] by inhibiting the phosphorylation of Ser498 of BACE1 and Thr654 of APP, altering the endocytic traffic of both proteins. However, direct inhibition of ROCK2 kinase activity would be a risky approach because of the diverse physiological roles of ROCK2 [42,43]. The recent approval of belumosudil, a highly selective, low-molecular-weight ROCK2 inhibitor against chronic graft-versus-host disease [44] also indicates the multiple functional roles of ROCK2, although a recent study [45] suggested that the therapeutic effectiveness of belumosudil was partially due to inhibition of the protein kinase CK2α. We believe that by avoiding side effects, pathway-specific inhibitors could provide a solution for the reduction in Aβ levels. This prompted us to study the molecular mechanism of the phosphorylation of BACE1 and APP by ROCK2 and map the protein–protein interactions involved.

Both BACE1 and APP are transmembrane proteins with short intracellular tails, while ROCK2 is an intracellular protein anchored to the membrane through its C-terminal regulatory domains. To avoid having to work with the full-sized proteins, we simplified the problem to those domains of the proteins which are in the same compartment, namely the C-terminal tails of BACE1 and APP, alongside ROCK2 kinase.

To understand the molecular mechanism of interaction between ROCK2 and its protein ligands, several lines of protein–protein interaction studies were undertaken. Our results clearly indicated the direct, although weak, interaction between ROCK2 and the C-terminal tail of BACE1. We could not detect a binary interaction between ROCK2 and APP_646–695_. However, in the presence of BACE1, high-affinity interactions of ROCK2 and APP_646–695_ were observed.

**Figure 5:**
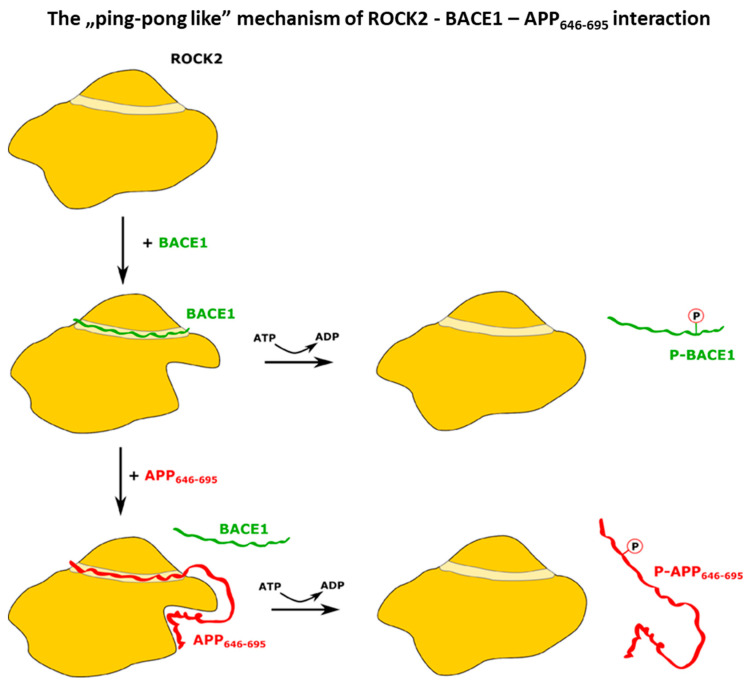
Scheme of the proposed ping-pong-like mechanism for the interaction and phosphorylation of BACE1 and APP by ROCK2 kinase. The kinase domain presents a canonical substrate-binding groove where the C-terminal peptide of BACE1 can bind. Upon the binding of BACE1, a conformational change occurs, creating a new allosteric binding site on ROCK2, which APP can interact with. The phosphorylation of BACE1 and the dissociation of p-BACE1 restores the initial state of ROCK2. When APP binds to the distorted ROCK2, BACE1 dissociates without the phosphorylation event but ROCK2 can phosphorylate APP. The dissociation of p-APP leads to the original ROCK2 conformation.

Based on the binding experiments, we constructed a sequential model (Figure 5) for the mechanism of ROCK2 action on BACE1 and APP. In this model, BACE1 recruits APP to ROCK2 for phosphorylation. In the native conformation of ROCK2, BACE1 binds in the binding groove of ROCK2 [46], and this binding event drives a conformational change in ROCK2, inducing a new allosteric binding site. ROCK2 can interact with APP_646–695_ in this new conformation through a dual binding site, where the newly formed allosteric site binds the C-terminal (665–695) region of APP_646–695_, while the binding groove of ROCK2 binds the N-terminal part, displacing BACE1. ROCK2 can now phosphorylate APP at Thr654, and phospho-APP dissociates, while ROCK2 is converted back to its original, native conformation. Alternatively, if APP is not available to displace BACE1 from ROCK2, ROCK2 can phosphorylate BACE1, which then dissociates.

This highly cooperative action between ROCK2, APP and BACE1 is in accordance with previous results indicating that the physical proximity of BACE1 and APP on the cell membrane is a main determining factor leading to the amyloidogenic pathway of APP processing [47,48].

Our results prove that the allosteric inhibition of the phosphorylation of APP by ROCK2 is a new, feasible option in the fight against Alzheimer’s disease. Site-directed mutagenesis experiments are in progress to localize and map the relevant orthosteric and allosteric sites.

## 4. Materials and Methods

### 4.1. Protein Expression and Purification

The synthetic gene (Geneart AG, Regensburg, Germany) of human APP_646–695_ was cloned into the pONE10K [49] vector between NheI and NotI cleavage sites, using primers 5′-GGGCTAGCGTGATGCTGAAGAAGAAACAG-3′ and 5′-GGGCGGCCGCCTAGCAGTTCTGCATCTGCTCAAA-3′. The APP_646–664_ construct was prepared using primers 5′-GCGCATATGAAAATCGAAGAAGGTAAACTGG-3′ and 5′-GCGGCGGCCGCCTAGTCAACCTCCACCACACCATGATG-3′, cloned between NdeI and NotI restriction sites. The APP_665–695_ truncated construct was constructed using the QuikChange Site-Directed Mutagenesis Kit (Agilent Technologies, Santa Clara, CA, USA) protocol using the following oligos: 5′CCTGTACTTCCAGGCCGCTGTCACCCC-3′ and 5′-GGGGTGACAGCGGCCTGGAAGTACAGG-3′. All primers used in this study were synthetized by Microsynth AG, Balgach, Switzerland.

The fusion protein constructs for the various APP peptides (containing maltose-binding protein (MBP) tag, which is a double-purpose expression tag, both enhancing protein expression and enabling amylose–resin-based affinity purification) [50] were transformed into Rosetta2 E. coli cells (Sigma-Aldrich, Burlington, MA, USA). The proteins were expressed at 37 °C in the presence of 50 µg/mL kanamycin and 30 µg/mL chloramphenicol. Protein expression was induced by adding 1 mM Isopropyl β-d-1-thiogalactopyranoside to the cell cultures upon reaching an OD_600_ density of ~0.6–0.8. Before induction, cultures were equilibrated to an induction temperature of 21 °C for 0.5 h. Protein expression was continued overnight at 21 °C and 220 rpm. The MBP-tagged proteins were purified using amylose affinity chromatography. The cells were suspended in 25 mM of HEPES with a pH of 7.4, 500 mM NaCl and 1 mM dithiothreitol (DTT). The bound proteins were eluted by a buffer containing 10 mM maltose. The collected protein fractions were applied ton a Superdex 75 gel filtration column equilibrated in 25 mM HEPES with a pH of 7.4, 150 mM NaCl and 1 mM DTT. Peak fractions were pooled and concentrated. The purity of proteins was tested using sodium dodecyl sulphate–polyacrylamide gel electrophoresis (SDS-PAGE) stained with Coomassie Brilliant Blue. All proteins were concentrated and stored at −20 °C.

The synthetic gene of human ROCK2 (1–1388) was cloned into the pONE30A vector between NheI and NotI cleavage sites. The ROCK2 kinase domain (27–417) construct (ROCK2-KD) truncation was prepared using primers 5′-GATCGCTAGCCAGAGGAAGCTGGAGGC-3′ and 5′-GATCGCGGCCGCTCTATAGTAGGTAAATCCGATG-3′ and cloned into the pONE30A vector between NdeI and NotI restriction sites. Both protein constructs (containing MBP tags at the N-terminus) were expressed in Sf9 insect cells after co-transfection using flashBAC GOLD baculoviral expression system (Oxford Expression Technologies, Oxford, UK). After virus amplification, insect cells at 2 × 10^6^ cell/mL density were transfected with the recombinant baculovirus to express the protein of interest in 500 mL Insect-XPress medium (Lonza, Basel, Switzerland) for 3 days at 27 °C in a shaker flask at 220 rpm. The proteins were purified using amylose affinity chromatography in 25 mM HEPES with a pH of 7.4, 500 mM NaCl and 1 mM DTT. The bound proteins were eluted by a buffer containing 10 mM maltose. The collected protein fractions were dialyzed and concentrated against 25 mM HEPES buffer (pH 7.4) [13].

### 4.2. Peptides

For the studies, the reporter peptide, BACE1 (QWCCLRCLRQQHDDFADDISLLK), both in free form and N-terminally labeled with 5-FAM (5-Carboxyfluorescein), was purchased from GenScript (Piscataway, NJ, USA).

### 4.3. Fluorescence Labeling

APP_646–695_ was labeled with TAMRA (T6027 Invitrogen, Waltham, MA, USA) according to the following procedure: 5-Carboxytetramethylrhodamine dissolved dye was added in 2-fold molar excess to a purified APP_646–695_ sample and then incubated in the dark for 2 h at room temperature. Unreacted dye was removed on Ultracel-10K centrifugal filters (Sigma-Aldrich, Burlington, MA, USA). For enzyme activity, the MBP tag was removed using tobacco etch virus protease cleavage. The cleavage was performed in a 20-time molar excess of TEV protease at room temperature for 2 h. The APP_646–695_-TAMRA constructs were separated from MBP using amylose affinity chromatography. The flowthrough was applied to a Superdex 75 gel filtration column equilibrated in 20 mM HEPES with a pH of 7.4, 150 mM NaCl and 1,5 mM EDTA. Peak fractions were pooled and concentrated. The purity of proteins was tested by SDS-PAGE stained with Coomassie Brilliant Blue and determined as ≥90% pure. The APP_646–695_-TAMRA was concentrated and stored at −20 °C.

### 4.4. Fluorescence Polarization

Changes in the FP signal in direct binding affinity measurements were monitored as a function of the increasing concentration of purified APP variants with a Synergy H4 (Agilent, Santa Clara, CA, USA) plate reader in 384-well plates. The labeled peptides were at 10nM in 20 mM Tris with a pH of 8.0, 100 mM NaCl, 0.05% Brij35P and 2 mM DTT. The affinities of the unlabeled peptides or different APP constructs to ROCK2 were measured in steady-state competition experiments, as follows: 50 nM (or 1 µM) labeled reporter BACE1 was mixed with ROCK2 in a concentration to achieve 25–50% complex formation. Subsequently, increasing amounts of unlabeled peptide or protein were added and the FP signal was measured as described earlier for direct affinity determination experiments. The dissociation constant (K_d_) was determined by fitting the data to the competition-binding Morrison equation [51] with GraphPad Prism 9 (GraphPad Software, San Diego, CA, USA). Titration experiments were carried out in duplicates, and the average FP signal was used for fitting the data.

### 4.5. Surface Plasmon Resonance (SPR)

SPR experiments were measured on a Biacore X (Cytiva, Danaher, Washington, DC, USA) two-channel SPR system using CM5 sensors. Full-length ROCK2 protein was immobilized using an Amine Coupling Kit. The surface matrices were activated by a 7 min injection of an aqueous solution of 0.2 M 1-ethyl-3-(3-dimethylpropyl)-carbodiimide and 0.05 M N-hydroxysuccinimide. Then ROCK2 at 50 μg/mL concentration in 0.01 M Na-acetate (pH 5) was injected into the sensor cell (30–60 μL). Remaining N-hydroxysuccinimide-ester groups were inactivated by an injection of 35 μL 0.1 M ethanolamine-HCl. Experiments were executed in an HBS-EP buffer (10 mM HEPES, 0.15 M NaCl, 3 mM EDTA and 0.005% Surfactant P20 (= Tween 20) with a pH of 7.4). During the analysis, the flow rate was 10 μL/min for both the association and dissociation phases. BACE1CD and APP were used at 7 μM, while MgATP was used at 100 μM concentrations.

### 4.6. Modeling of the Binary Complexes

Structures of ROCK2-BACE1 and ROCK2-APP_646–695_ complexes were predicted using AlphaFold-Multimer [39] as implemented in ColabFold [40]. Version 1.3.0 of ColabFold was run locally as implemented in LocalColabFold (https://github.com/YoshitakaMo/localcolabfold, accessed on 26 September 2022). The number of recycling iterations was set to 3. Five models were generated, and the best model, as ranked by the interface pTM score, was selected. Models were built with both the homodimeric and monomeric forms of ROCK2. Since no interaction was found between the substrate (BACE1 or APP_646–695_) and the distant ROCK2 subunit, we present the models with the monomeric ROCK2 in this paper.

## Data Availability

All datasets generated for this study are included in this article.
